# How Can Microarrays Unlock Asthma?

**DOI:** 10.1155/2012/241314

**Published:** 2012-02-06

**Authors:** Alen Faiz, Janette K. Burgess

**Affiliations:** ^1^Woolcock Institute of Medical Research, 431 Glebe Point Road, Glebe, NSW 2037, Australia; ^2^Central Clinical School, The University of Sydney, Sydney, NSW 2006, Australia; ^3^Department of Pharmacology, The University of Sydney, Sydney, NSW 2006, Australia; ^4^Cooperative Research Centre for Asthma and Airways, Glebe, NSW 2037, Australia

## Abstract

Asthma is a complex disease regulated by the interplay of a large number of underlying mechanisms which contribute to the overall pathology. Despite various breakthroughs identifying genes related to asthma, our understanding of the importance of the genetic background remains limited. Although current therapies for asthma are relatively effective, subpopulations of asthmatics do not respond to these regimens. By unlocking the role of these underlying mechanisms, a source of novel and more effective treatments may be identified. In the new age of high-throughput technologies, gene-expression microarrays provide a quick and effective method of identifying novel genes and pathways, which would be impossible to discover using an individual gene screening approach. In this review we follow the history of expression microarray technologies and describe their contributions to advancing our current knowledge and understanding of asthma pathology.

## 1. Introduction

Asthma is a complex chronic inflammatory disease which affects ~300 million individuals worldwide, causing an estimated economic cost of $19.7 billion in direct and indirect costs each year [1, 2]. Asthma can be defined by a number of characteristics, including (1) airway hyperresponsiveness (AHR), (2) airway remodeling, and (3) airflow obstruction including bronchoconstriction, mucus plugging, and inflammation [3]. The presence and severity of these characteristics can be influenced by many factors including age, ethnicity, gender, genetic predisposition, and the environment [4–7]. The asthma phenotype is further confounded by the existence of possible subtypes of asthma, which go beyond the common mild, moderate, and severe groupings [8]. This heterogeneity has thus far been a major hindrance in the search for susceptible genes for asthma, and it is becoming increasingly apparent that asthma is the result of dysregulation of a number of complex pathways instead of any single gene. In a new age of high-throughput technologies, gene-expression microarrays provide a quick and effective method of identifying novel genes and pathways which would be impossible to discover using an individual gene screening approach. Programs used to analyse and identify significant pathways based on microarray data have previously been reviewed [9] and will not be discussed here. In this review we will follow the history of gene-expression microarray technologies and describe their contributions to our current understanding of asthma pathology.

## 2. Methods for Identifying Disease Causing Genes

Since early evidence for a genetic component for asthma was most strongly demonstrated by a higher concordance for asthma among monozygotic than dizygotic twins [10], the search for genes influencing this disease has relied on three main approaches: genomewide association studies (GWASs)/locus fine mapping, gene candidate approaches, and gene expression studies (gene-expression microarrays). The first two methods have been extensively reviewed [11, 12] and therefore will only be briefly mentioned here. GWASs have been essential in the discovery of many asthma-associated genes including disintegrin and metalloproteinase domain-containing protein 33 (ADAM33) (the extensively studied gene thought to be involved with airway remodeling), inactive dipeptidyl peptidase 10 (DPP10), neuropeptide S receptor 1 (NPSR1), histocompatibility antigen, class I, G (HLA-G), and PHD finger protein 11 (PHF11) [12–16]. GWASs rely on the variation of genes or surrounding DNA which occurs between individuals and uses this variation to measure the probability that certain single nucleotide polymorphisms (SNPs) (changes to the DNA sequence which may result in changes to the amino acid sequence of a protein) are linked to a disease. Because no prior knowledge of gene function is required, GWASs are considered an unbiased technique. In contrast, the gene candidate approach only looks at a specific region of the genome within or surrounding a gene of interest.

Gene-expression microarrays provide a platform to measure and compare the expression level of all genes within a genome at a single point in time. This platform therefore allows users to identify genes/microRNAs (miRNAs) which may be up/downregulated when comparing different types of tissue (e.g., diseased versus normal) or stimulations with certain drugs (treated versus untreated). Like GWAS, gene-expression microarrays are considered an unbiased technique allowing for the identification of truly novel genes. Furthermore gene-expression microarrays provide a tool to genetically profile diseases, helping to separate diseases into subtypes or predict the outcome of certain treatments. Despite numerous advantages, the use of gene-expression microarrays in asthma research is still in its infancy.

## 3. Macroarrays: Where It All Began

**Figure 1:**
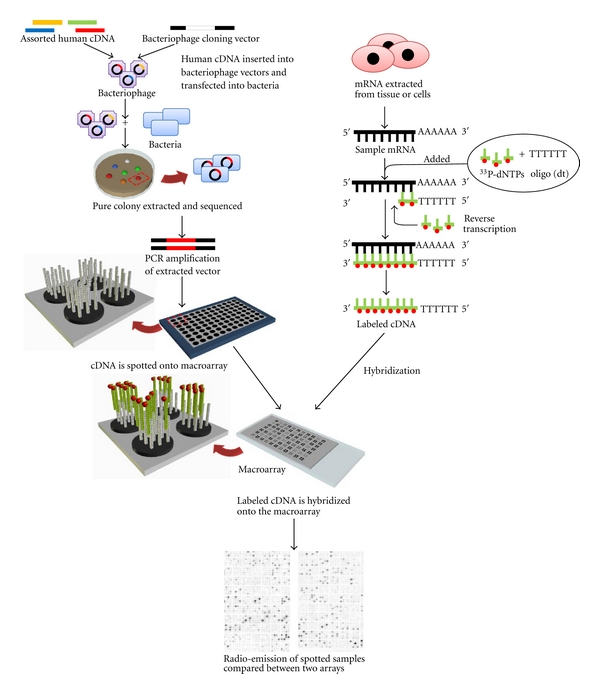
Overview of the production and use of Macroarrays. Macroarrays were constructed from cDNA held within bacterial libraries. These libraries were developed by inserting total human cDNA into bacteriophage vectors and transfection into bacteria. Pure colonies of bacteria carrying vectors were sequenced and amplified by PCR prior to spotting on to a macroarray. Samples were labeled by reverse-transcribing mRNA with radioactively labeled ^33^phosphate-deoxyribonucleotide triphosphates (^33^P-dNTPs) using specific oligo(dT) primers. Labeled cDNA samples were hybridized to duplicate macroarrays where gene expression was quantified by comparing the radio-emissions of each spot.

Macroarrays were the predecessors to the current day gene-expression microarray; they had the ability to test anywhere between 500 and 18000 cDNA transcripts, which were usually spotted onto a nylon membrane by an arrayer (a device connected to a computer allowing for precise placing and cataloguing of samples on an array) (Figure 1) [17]. The cDNA spotted onto macroarrays was obtained from bacterial libraries, which were developed by inserting total human transcripts into bacteriophage vectors and transfecting these vectors into bacteria, usually *Escherichia coli*. These vector carrying bacteria were grown and pure colonies were sequenced and amplified by PCR prior to being spotted on a macroarray.

Target transcripts for macroarrays were usually radioactively labeled by reverse-transcribing sample RNA with ^33^phosphate-deoxyribonucleotide triphosphates (^33^P-dNTPs). Samples were then hybridized to the spotted macroarray and quantified by measuring the amount of radio-emission from each spot. Differential gene expression was calculated by comparing the emission intensity of samples spotted on to duplicate macroarrays. Despite being the ground-breaking technology of their day, macroarrays had a number of problems. The main limitations of macroarrays were the low density of probes per array (fewer genes could be investigated per array), the large volumes of sample required for hybridization (up to 50 mL compared with 200 *μ*L used for current gene-expression microarrays), and the reliability of the bacterial libraries. In some cases the bacterial libraries were not composed of pure colonies (not all bacteria in a single spot contained the same cDNA insert) making it difficult to determine which transcript was represented by a particular spot on the macroarray.

## 4. Gene-Expression Microarrays

**Figure 2:**
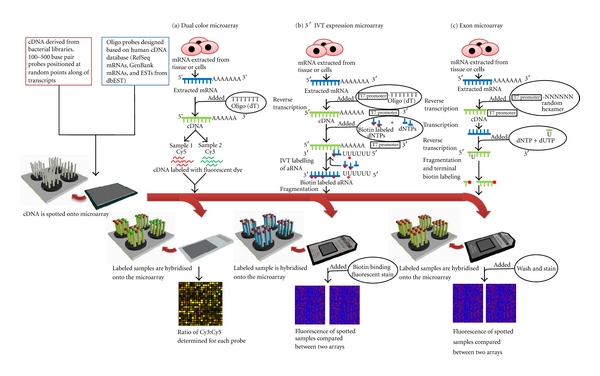
Overview of the production and use of expression microarrays. 3′ Expression arrays use synthetically derived oligo probes with design based on mRNA Databases (RefSeq mRNAs, GenBank mRNAs, and ESTs from dbEST) or cDNA derived from bacterial libraries (see Figure 1). Sample mRNA can be labeled using two methods (a) Cy3/Cy5 labeling: sample mRNA is reverse transcribed into cDNA and Cy3 is added to one sample and Cy5 to another. Both labeled samples are hybridized to the same microarray. (b) 3′ IVT array: sample mRNA is reverse transcribed to cDNA using oligo(dT) primers, to provide a template for transcription. Using biotin-conjugated nucleotides, the template cDNA is then converted to amplified RNA (aRNA). The biotin-labeled aRNA samples are then fragmented and hybridized onto 3′ expression arrays. A biotin binding fluorescent stain is added to the microarray after hybridization. (c) Affymetrix HuExon 1.0 ST: sample mRNA is reverse transcribed to cDNA using random primers, to provide a template for transcription. The resulting RNA is then reverse transcribed in the presence of dUTPs which are incorporated occasionally into the cDNA sequence instead of dTTP. An enzyme is then used to cleave the cDNA at the site of dUTP incorporation and fragments are terminally labeled before hybridization onto the array. The microarray is then washed and stained after hybridization.

Microarrays were the next step forward in the evolution of gene expression studies, with the advances in array technology being pioneered by Patrick Brown's laboratory [18]. Microarrays, unlike their predecessor, were spotted onto glass slides allowing for a higher density of probes (decreasing the amount of sample required to interrogate the same number of genes) and no longer used radio-actively labeled nucleotides. These arrays were created using a precise *xyz* robot that was programmed to spot cDNA samples on the substrate in precise locations to allow identification of genes with expression changes during the analysis phase of the experiment [18]. A number of technologies have been released using this platform including the dual color microarray (or two-color microarray) process explained in Figure 2 and these have been reviewed previously [17]. An alternative technology for the production of microarrays was developed using photolithographic masks to create templates to enable *in situ* synthesis of oligonucleotides (usually 20–30 bps) directly on the glass substrate. Affymetrix pioneered the use of this platform of array production with the development of their “GeneChip” series of arrays, and in this review we will focus on the 3′  *in vitro* transcription (IVT) Expression GeneChip, as the majority of asthma-related studies have been conducted using this platform; however there are a large range of other expression microarrays produced by other companies which have been previously reviewed [19]. 

## 5. 3′ IVT Expression Microarrays

The 3′ IVT array microarray is historically the most common platform used by researchers conducting gene-expression microarray experiments in the asthma research field. The initial asthma gene-expression microarray studies using human cells were conducted in 2001 on the Affymetrix Hugene FL microarray containing probes representing ~6,500 human genes from the UniGene Build 18, GenBank, and the Institute for Genomic Research (TIGR) databases (Table 1). As gene-expression microarray technology advanced and mRNA databases became more complete, further versions of this platform were released, increasing the number, specificity, and annotation of the microarray probes with each subsequent release (Table 1). In 2004, the asthma community turned to the Affymetrix GeneChip 95A, the successor for the Affymetrix Hugene FL microarray, containing probes for ~12,000 full-length genes, derived from sequences in UniGene Build 95A (created from GenBank 113 and dbEST/10-02-99), including all the sequences represented on the Hugene FL microarray (Table 1).

In recent years, Affymetrix has released the Affymetrix GeneChip Human Genome U133 (HG-U133) containing probes representing ~33,000 genes (created from GenBank, dbEST, and RefSeq) followed by their most recent version, the Affymetrix GeneChip Human Genome U133 Plus 2.0 array, which contains all the probes from its previous version plus those for 6,500 new genes (Table 1).

### 5.1. Preparing Samples for Analysis on the 3′ IVT Expression GeneChip

**Figure 3:**
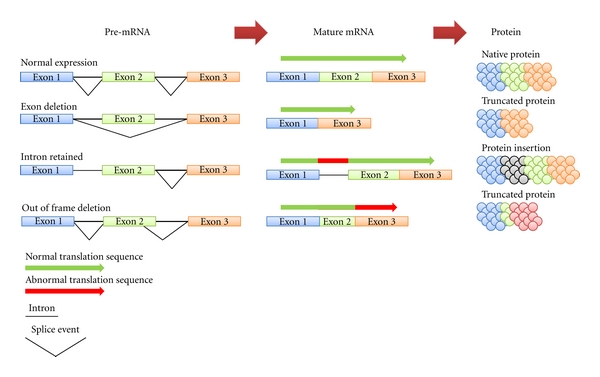
Transcripts from a single gene can undergo different splicing events. When mRNA is initially transcribed (known as pre-mRNA), it retains introns (thick black line), large segments of noncoding mRNA which separate exons, the coding regions. Through a process known as splicing, the introns are then removed and exons are ligated together to produce mature mRNA. Splicing also has the ability to remove exons or even retain introns resulting in the formation of different mature mRNA transcripts for the same gene (referred to as alternative splicing). Different mature mRNA transcripts encode for different proteins which may have alternative functions.

Although many versions of the 3′ IVT array have been released, the methods for preparing samples for these microarrays remain mostly unchanged. To prepare the samples for the 3′ IVT array, mRNA is first extracted from the targeted sample and converted to cDNA via reverse transcription using oligo(dT) primers attached to a T7 promoter (Figure 3). Oligo(dT) primers are short strings of dTs which selectively bind to the poly-A tails (of mRNA). Although this process was quite successful in binding to the majority of mRNAs, transcripts without poly-A tails (non-polyadenylated) were lost during this step. Current technology for the purpose of priming for reverse transcription uses random hexamers (strings of six random dNTPs) which capture sequences at any location along a transcript. This will be discussed later (see Section 8). 

The cDNA is then converted to double stranded DNA (using the T7 promoter), to provide a template for transcription. Using biotin-conjugated nucleotides, the template DNA is then converted to amplified RNA (aRNA). The biotin-labeled aRNA samples are then fragmented and hybridized onto 3′ expression arrays and visualized by staining with phycoerythrin.

### 5.2. 3′ IVT Expression GeneChip Probes

Unlike the macroarrays previously described and a number of other gene-expression microarrays available on the market, 3′ IVT Expression GeneChips do not use cDNA libraries spotted onto an array. Instead Affymetrix arrays use short (~25 bp) nucleotide probes synthesized directly on the array; this process is well explained in a previous review [17]. Gene expression is determined by the hybridization of transcripts to perfect match (PM) and mismatch (MM) probes. Transcripts will preferentially bind to PM probes as they provide a perfect complimentary sequence to their matching transcript. MM probes are designed to resemble PM probes but differ (change in a single nucleotide) just enough for the target transcript not to bind. Therefore any transcripts binding to these MM probes are considered to represent background hybridization; by the use of the function (PM hybridization, MM hybridization) background hybridization can be calculated and taken into account. However, the use of MM probes to identify background binding has been slowly phased out because of a variety of technical reasons including the occurrence of “negative” expression levels when expression is low and the MM intensity exceeds the PM. For example, the R-Bioconductor preprocessing pipelines frequently omit MM probes [20].

## 6. 3′ Expression Arrays: Influence on Asthma Research

3′ Expression arrays have played a key role in asthma research through the screening for, and identification of, genes which are affected by asthma relevant stimuli and the direct comparison of asthmatic tissue to nonasthmatic tissue. Initial studies for asthma using the 3′ platform focused on identifying key cell types which play a role in asthma; therefore many researchers conducted their studies on human-isolated cells expanded in culture. One of the first isolated cell gene-expression microarray studies was conducted by Lee and group in 2001, where commercially available primary airway smooth muscle (ASM), epithelial cells, and fibroblasts derived from human lungs were treated with 100 ng/mL of interleukin 13 (IL13), a cytokine known to be upregulated in asthma, for 6 hours and run on an Affymetrix Hugene FL microarray [21]. This study identified that treatment with IL13 caused dysregulation of a number of asthma-related genes. Differing effects were observed in different cell types of the airway, promoting the idea that each cell type plays its own role in asthma [21]. Once the ball started rolling, the asthma gene-expression microarray field quickly expanded from looking at single treatments on pure isolated cells in culture to the effects of more complex interactions including genes expressed during viral infection and direct comparisons of the gene expression in asthmatic and nonasthmatic tissue [22]. Although there have been many murine gene-expression microarray studies analyzing models of asthma, we have focused on the human studies in this review.

### 6.1. Airway Smooth Muscle Cells

The ASM plays a key role in the normal constriction and relaxation of the bronchial airway. In asthma the role of the ASM becomes exaggerated resulting in excessive airway narrowing in response to nonantigenic stimuli, termed AHR. A number of factors have been implicated in promoting AHR including airway remodeling and inflammation. The majority of the ASM gene-expression microarray studies to date have focused on the latter parameter and most focusing on the effect of IL13 [21, 23–25] but a small number have been conducted on remodeling [26]. In the search for an inflammatory mediator for asthma, IL-13 was found to play a critical role in murine asthma models [27]. During this time microarrays were just starting to be used in asthma research and many researchers took advantage of this new screening technology to help identify genes stimulated by this inflammatory cytokine. As already discussed the first of these studies was conducted by Lee et al., who identified a number of genes which were expressed specifically by ASM after treatment with 100 ng/mL of IL13 for 6 hours [21]. The next major study was conducted by Jarai et al. in 2004, who again looked at the effect IL13 had on ASM cells and two additional treatments, interleukin-1*β* (IL1*β*) and transforming growth factor-*β* (TGF*β*) selected to identify if different inflammatory conditions cause ASM cells to distinct phenotype changes. Jarai et al. conducted this study using the updated Affymetrix GeneChip 95A array and stimulated ASM cells separately with 10 ng/mL of each treatment for 4 and 24 hours [23]. Although these authors conducted this study using cells derived from only two patients, a large range of genes induced by these three stimuli were identified [23]. IL1*β* stimulation confirmed the induction of a number of cytokines found in the literature to be previously upregulated; also a number of novel genes were identified including growth-related oncogene *α*, *β*, and *γ*, macrophage inflammatory protein 3*α* (MIP-3*α*), epithelial neutrophil activating peptide 78 (ENA-78), granulocyte-colony stimulating factor (G-CSF), and interleukin-1 receptor antagonist (ILRA) [23]. The main effect of IL13 stimulation on ASM was the induction of the expression of eotaxin, a strong chemoattractant for Th2-like T lymphocytes basophils and eosinophils which are found in tissues undergoing allergic reactions [28]. A note made by the authors was that this gene was not previously picked up by Lee et al. in their gene-expression microarray study and this disparity was thought to be due to differences in the concentration of IL13 and the sources of the ASM cells. TGF*β* altered the expression of a number of structural and extracellular matrix proteins and also increased expression of IGF-binding protein 2, which had previously been indicated to mediate the growth response of TGF*β* on ASM cells [23, 29].

High serum immunoglobulin E (IgE) levels have long been associated with allergic asthma [30]. In 2000 an association study identified a naturally occurring isoform of IL13 (IL13R130Q) to be associated with elevated serum IgE levels [27]. In an attempt to identify the role of IL13 and its isotypes in the pathogenesis of allergic asthma, Syed et al. looked at the effect of IL13 and IL13R130Q on ASM using an expression microarray containing 8159 human gene cDNA clones from Research Genetics (IMAGE consortium, Huntsville, AL), Incyte Genomics (Santa Clara, CA) [24]. No differences were detected between the genes induced by the two isoforms of IL13; however two genes, vascular cell adhesion protein 1 (VCAM1) and IL13 receptor alpha 2 protein chain (IL-13R*α*2), were identified as being stimulated at both the mRNA and protein level [24]. VCAM1 had previously been implicated as a key player in the inflammation process; therefore the microarray further validated the role of IL13 in asthma [24]. IL13 induction of IL-13R*α*2 was predicted by the author and that newly synthesized IL-13R*α*2 may act as a decoy receptor therefore creating a self-regulating feedback loop preventing overstimulation of ASM cells, which had been previously confirmed in murine models [31].

### 6.2. Airway Epithelial Cells

The airway epithelium lies on the outermost layer of the bronchus and hence is positioned to directly respond to environmental irritants such as pollutants and viruses which are associated with asthmatic exacerbations. Previous studies have shown that asthma epithelium has alternations in both its structure and gene expression [32, 33]. The majority of gene-expression microarray studies focusing on the structural cells of the airway have focused on the epithelium [34–42]. Following the initial studies conducted by Lee et al., Yuyama et al. looked at the effect of Th-2 cytokines on human bronchial epithelial cells (*n* = 3) by treating them with 10 ng/mL of interleukin-4 (IL-4) and 50 ng/mL of IL13 separately for 24 hours before running the resulting cDNA on a Affymetrix Hugene FL microarray [34]. This study identified 2 major genes—squamous cell carcinoma antigen 1 (SCCA1) and squamous cell carcinoma antigen 2 (SCCA2) which were both increased by ~20 fold in both stimulations compared to untreated cells. SCCA1 and SCCA2 expression was found to be under the control of signal transducer and activator of transcription 4 (STAT4), a transcription factor previously found to be upregulated in epithelial cells derived from severe asthmatics [34]. Furthermore SCCA1 was found to be upregulated in the serum of asthmatic patients especially during an asthma exacerbation [34]. Recently both SCCA1 and SCCA2 have been proposed as a method for testing for bronchial asthma attacks through the use of serum samples or mRNA expression [43].

In 2003 Kong et al. looked at early gene expression during a respiratory syncytial virus (RSV) infection of A549 epithelial cells using an Affymetrix Hugene FL microarray [35]. They found that two pathways, signal transducer and activator of transcription 1*α* and 3 (STAT-1*α* and STAT-3), were upregulated by RSV-key genes required for successful infection [44]. Subsequently the microarray identified 53 genes which had a change in expression due to RSV infection, consistent with changes in gene expression reported in previous studies [44].

Taking a different approach, Chu et al. looked at the effect of mechanical stress on gene expression in epithelial cells [36]. During bronchial constriction the epithelial layer experiences compressive mechanical stress [45], which in previous studies have been shown to stimulate the expression of transforming growth factor-*β* 2 (TGFB2) and endothelin (ET) facilitating fibrosis of the airway wall, a feature of asthmatic airways [46]. To identify further genes affected by mechanical stress, Chu et al. placed epithelial cells under mechanical stress for 8 hours and ran the samples against a set of pooled controls on Affymetrix Human 133A DNA microarrays [36]. Chu et al. identified a number of plasminogen-related genes (urokinase plasminogen activator (uPA), urokinase plasminogen activator receptor (uPAR), plasminogen activator inhibitor-1 (PAI-1), and tissue plasminogen activator (tPA)) which were upregulated on the microarray and confirmed both by qPCR and at the protein level [36]. Activation of the plasminogen pathways was shown to activate MMP-9, a protein associated with airway remodeling [47]. These results support the growing body of evidence that noninflammatory stimuli can contribute to the overall asthma phenotype [36].

In recent years two groups have conducted large screening for genes differentially expressed in asthma epithelium [41, 42]. The first of these two studies was conducted in 2007 by Woodruff et al. who compared the gene expression of 42 asthmatics and 44 nonasthmatics (28 healthy subjects and 16 smokers) and also the effect of corticosteroids on asthmatic patients using an Affymetrix Human 133A 2 plus microarray. The authors identified 22 genes which were found to be differentially expressed between asthma and healthy controls including periostin and serpinB2 and were verified by qPCR and at the protein level. The use of corticosteroids in asthmatic patients was shown to affect 30 genes compared to a placebo; 5 were verified by qPCR, including FK506 binding protein 51 (FKBP51), which had previously been identified to modulate glucocorticoid receptor activity [48].

The second study conducted in 2010 by Kicic et al. looked at the expression of children with asthma (*n* = 36), healthy atopic (*n* = 23) and healthy nonatopic controls (*n* = 53) using Affymetrix Human 133A DNA microarrays [42]. The aim of the study was to identify extracellular matrix (ECM) protein differentially expressed in asthma. They identified a single ECM gene fibronectin (FN) which was significantly lower in asthmatics, verified by qPCR and ELISA [42]. The authors then silenced FN expression in nonasthmatic epithelial cells and this was found to inhibit wound repair, while in the reverse situation the addition of FN to asthmatic epithelial cells restored wound repair within these cells [42]. Based on these results FN was identified as an ECM protein which may contribute to the abnormal epithelial repair seen in asthmatics.

### 6.3. Fibroblasts

Currently no further asthma-related gene-expression microarrays' studies have been conducted on human lung fibroblasts following the initial study conducted by Lee et al. [49]; however a number of arrays have been conducted on lung fibroblasts from murine models which have been reviewed elsewhere [50].

### 6.4. Mast Cells

Mast cells play an important role in asthma and other allergic disorders. Activation of mast cells by stimulatory factors, such as antigens or IgE, induces the production and/or release of cytokines and inflammatory mediators such as histamine. The use of gene-expression microarrays for human mast cell studies has been limited because of the difficulty of isolating this cell type [51–53]. The initial gene-expression microarray studies conducted on mast cells aimed to identify genes which were specifically expressed by mast cells. Nakajima et al. compared the expression of peripheral blood-derived mast cells, eosinophils, neutrophils, and mononuclear cells on an Affymetrix GeneChip 95A array [51]. They identified 140 genes which were mast cell specific and major basic protein (MBP) which were thought to be eosinophil specific. Furthermore MBP protein expression was verified by flow cytometry and confocal laser scanning microscopy. MBP is thought to be physiologically important in asthma as it has previously been found to be deposited in the damaged epithelium of asthmatic patients [54, 55].

### 6.5. Tissue

It has long been recognized that smooth muscle cells expanded in culture lose their contractile function and receptors with subsequent passaging [56]. The loss of function has also been noted in other cell types of the human airways. Therefore, the question has been raised as to whether gene-expression microarray studies on cultured cells give a true representation of physiological conditions. Tissue-based studies therefore provide a glimpse of the genes expressed under true physiological conditions, but because this source of mRNA is a mixture of cell types, it is impossible to differentiate which transcripts are being expressed by which cell type. One of the few tissue microarray expression studies, and the first 3′ microarray study to directly compare human asthmatic and nonasthmatic tissue, was conducted by Laprise et al. looking at the expression profile of biopsies before and after inhaled corticosteroid therapy [22]. Using an Affymetrix GeneChip 95A, Laprise et al. identified 74 genes which were differentially expressed between asthmatics and nonasthmatics, with a majority of these genes having already been identified as asthma related. However a number of novel genes were also identified including arachidonate 15-lipoxygenase (ALOX15), cathepsin C (CTSC), and chemokine (C-X3-C motif) receptor 1 (CX3CR1) [22]. Comparing asthmatic subjects before and after inhaled corticosteroid therapy identified 128 genes which had altered expression in the presence of the therapy. However 70% of the genes which were upregulated in asthma remained unchanged after corticosteroid therapy [22]. It was predicted by the author that a subset of these genes may be responsible for asthma chronicity [22].

However 3′ arrays only provide the user with the overall level of gene expression without measuring the degree of contribution of different splice variants of the genes being interrogated to the total gene expression. This is problematic, as a number of key asthma-related genes including NPSR1, IL-4, cytochrome c oxidase assembly homolog (yeast) (COX-11), and ADAM33 have been found to have dysregulation of alternative splicing patterns and/or differential expression of specific splice variants [15, 57–60].

## 7. Alternative Splicing

The human genome contains ~30,000 genes; however it has been predicted that there are over 100,000 proteins expressed in the body. These predictions are in contrast to the previously well-accepted “one gene-one enzyme” theory proposed in 1941, where it was believed that each gene encoded only one protein [61]. Recently, alternative splicing has been identified as the process through which this apparent gene deficiency in the human genome is explained. When mRNA is initially transcribed (known as pre-mRNA), it retains introns, large segments of noncoding mRNA which separate exons, the coding regions (Figure 3). Through a process known as splicing, the introns are then removed and exons are ligated together to produce mature mRNA sequences. Splicing also has the ability to remove exons or even retain introns resulting in the formation of different mature mRNA transcripts for the same gene (referred to as alternative splicing).

It is now predicted that over 95% of all multiexon genes in the human genome undergo some degree of alternative splicing [62]. Depending on what sections of RNA are removed or retained, alternative splicing can have major effects on the functionality of the resultant proteins (Figure 3). Therefore, it is not surprising that a number of genetic diseases including asthma have been linked with mutation and changes with cis- (e.g., DNA sequence related) and trans- (e.g., transcription factors) acting factors which lead to aberrant splicing and irregular protein production (Table 2).

The function of disease causing splice variants is still poorly understood; however, based on previous findings (Table 2), studying splice variants may provide an untapped resource which could hold some answers for their role in asthma.

## 8. The Future of Gene-Expression Microarrays: Affymetrix HuExon 1.0 ST

Recent advances in gene-expression microarray design have produced the Affymetrix HuExon 1.0 ST; this platform allows for the evaluation of not only gene expression but also exon level expression and identification of unknown splicing events. The Affymetrix HuExon 1.0 ST contains 65 million probes, 8 times the number of the probes present in the latest release of the Affymetrix U133 Plus2 array. The main differences between exon arrays and 3′ arrays come from the number and binding sites of the oligonucleotide probes, labeling methods and differing methods for identifying background noise levels.

3′ arrays simply use 11–20 probes for each gene which bind to the 3′ end, while Exon arrays use ~40 probes evenly spaced along all exons of a given transcript. The advantage of this method is that Exon arrays can detect all isoforms of a gene transcript and evaluate the level of expression for each splice variant, while 3′ arrays lack this ability as their probes are only positioned towards the 3′ end of an mRNA transcript.

Another key difference is the generation of the sample mRNA during the initial cDNA step; Exon arrays use random probes (containing 6 random nucleotides) attached to a T7 promoter whereas oligo(dT)s attached to a T7 promoter are used as the primers for the 3′ arrays. By using random probes which bind anywhere on the mRNA transcript (not restricted to the poly-A tail), Exon arrays have overcome the problem of identifying non-polyadenylated transcripts by covering the entire gene transcript, rather than having a 3′ bias. Replacing the function of the MM probe control used by 3′ arrays, Exon arrays use a specially designed set of probes which should not bind any mRNA to detect background binding. Exon arrays represent many improvements when compared to their predecessors and, as the search for disease causing candidates moves forward, it will only be a matter of time before these arrays receive much greater attention in the scientific community.

### 8.1. Affymetrix HuGene 1.0 ST Microarray

The Gene 1.0 ST Array was designed based on the Exon 1.0 ST Array (and therefore uses the same sample preparation and labeling methods). The key difference is that it contains only a subset of the probes, focusing on well-annotated content. Similar to 3′ arrays, the Affymetrix Gene 1.0 ST also provides a platform for measuring genomewide gene expression of a sample at a single point in time. However, by using probes which are evenly spaced along all exons, the Affymetrix Gene 1.0 ST array is able to give a true representation of gene expression (Figure 2). Gene 1.0 ST arrays also have a limited ability to identify alternatively spliced gene products; however the low number of probes per exon means that false positive events occur more commonly than with the superior Human Exon 1.0 ST Array.

### 8.2. The Influence of Exon Array Asthma Research

At the time of writing this review the use of exon arrays in reported human asthma-related projects was limited to a single study conducted by Plager et al. 2010 [63]. This study aimed to identify genes related to asthmatic chronic rhinosinusitis with nasal polyps (aCRSwNP) and eosinophilic epithelial inflammation, through the use of an Affymetrix HuExon 1.0 ST. Although alternative splicing was not looked at in this study, Plager et al. identified a number of chemokines and chemoattractants including eotaxin-1 (associated with inflammation and upregulated by IL13 [23, 28]), -2, and -3 which were associated with aCRSwNP.

## 9. MicroRNA

MicroRNAs (miRNA) are short (22 nucleotide) segments of RNA which bind to complementary sequences on target mRNA, thereby facilitating mRNA degradation and thus repressing gene expression at the transcriptional level. miRNAs can be transcribed from their own genes or exist within intronic regions of mRNA. miRNAs are incorporated into miRNA-argonaute complexes which facilitate their ability to degrade/inhibit mRNA transcripts (reviewed in [64]). The human genome is believed to encode over 1000 miRNAs [65], and these miRNAs are predicted to bind to over 60% of all mRNA transcripts in the human genome [66]. The dysregulation of miRNAs has been identified in a number of human diseases including asthma [67]. 

## 10. MiRNA Microarray: How It Works

**Figure 4:**
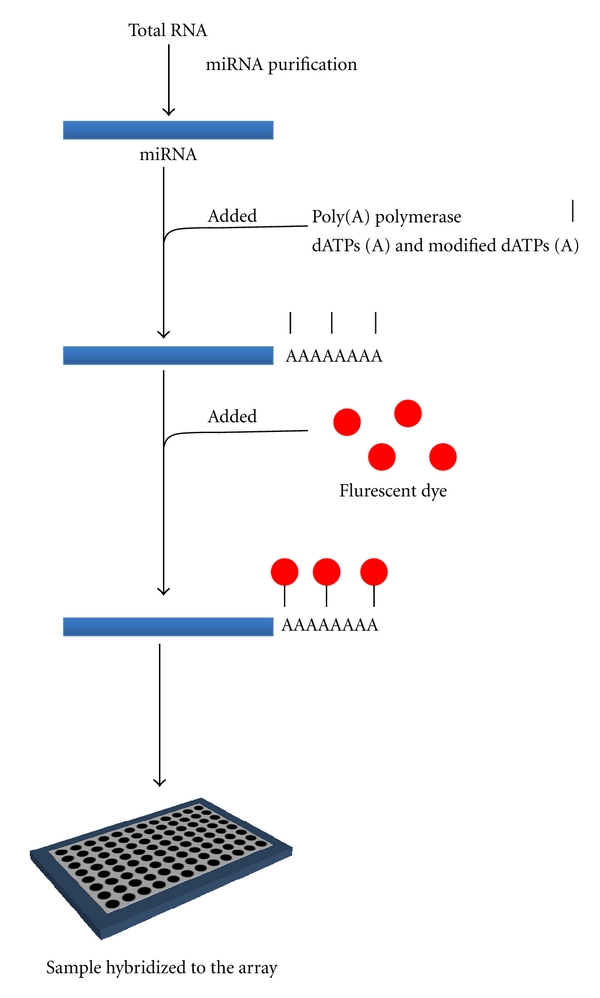
Overview of *mir*Vana miRNA bioarray methodology. Total RNA is extracted from tissue or cells and miRNA purified. Poly(A) polymerase is then added in the presence of modified dATPs and normal dATP. A poly(A) tail containing the modified dATPs is then added to all RNAs present in the sample. Fluorescent dye is added which binds to the poly(A) tail and the sample is hybridized to the array.

There are a large range of miRNA microarrays currently being offered by a number of companies (Table 3); however, as yet, the methodology used to analyze these arrays has not been standardized. In this review we will discuss the methods used by the *mir*Vana miRNA bioarrays V2 (Ambion) which were used by Kuhn et al. [67], currently the only miRNA array conducted on human ASM cells (Figure 4). The *mir*Vana miRNA bioarray, like a number of other miRNA microarrays on the market, relies on the addition of a poly (A) tail containing modified adenosine nucleotides to all remaining RNA (after miRNA purification) which allows for the specific binding of fluorescent dyes, prior to being hybridized to the array.

miRNA asthma research is still in its infancy, with the initial report of a human asthma miRNA array study being made by Kuhn et al. in 2010 [67]. These researchers looked at the effect of IL-1*β*, TNF-*α*, and IFN-*γ* on miRNA expression in ASM cells using *mir*Vana miRNA bioarrays V2 (Ambion). miR-25, miR-140*, miR-188, and miR-320 were found to be significantly downregulated, and these data were verified by both microarray and quantitative PCR. Furthermore miR-25 had previously been identified to regulate Krüppel-like factor 4 (KLF4), a mediator of proinflammatory signaling in macrophages [68]. Kuhn et al. confirmed that the downregulation of miR-25 allowed for the upregulation of KLF4 [67].

The role of miRNAs in asthma is still under investigation; however by identifying the role of certain miRNAs by up/downregulation of its expression and measuring its effects on overall gene expression (microarray) or using prediction software to predict genes which the miRNA may bind to and following this up by Real time PCR, researchers are slowly identifying the function of particular miRNA. In the future, miRNA may provide a source of novel treatments and therapies.

## 11. Challenges for Gene-Expression Microarray Projects

Despite numerous advantages, the use of microarrays still has many limitations, mainly relating to the experimental design, sample variance, and the statistics used to analyze the accumulated data. The challenges of microarray statistics in complex diseases have previously been extensively reviewed [69] and will not be discussed here.

### 11.1. Experimental Design

One of the main limitations of microarray studies today is still the cost; however this is slowly decreasing. In the past, cost was a major burden, often limiting the number of patients/samples analyzed in previous microarray studies; Jarai et al. and Yuyama et al. looked at the effect of Th-2 cytokines on human bronchial epithelial cells (*n* = 3) and primary ASM cells (*n* = 2) [23, 34]. Another contributing factor to the lack of patients analyzed is the concept of replicates versus treatments; when dealing with a limited number of arrays a decision one must make is whether to sacrifice the number of replicates to allow for an increase in the number of treatments studied, or vice versa. Traditionally, when dealing with different treatments of the same cell type which encompass the majority of asthma-related microarray studies, studies were designed to increase the number of treatments. The low patient/sample per treatments number was then overcome by conducting followup experiments such as Quantitative Real-time PCR and/or ELISA with a greater patient pool on single candidate genes [23, 34]. However, this still leaves an extensive list of genes to followup which is usually impractical with these alternative methods.

### 11.2. Sample Variance

Factors such as patient-to-patient variation and the heterogeneity of the asthma phenotype can make microarray data unreliable and hard to replicate. Therefore, it is important that the right type and number of patients are selected for each study, hence reducing the variation within the samples. A problem that many asthma studies face is obtaining samples with similar asthma diagnoses. Using patients who have the same level of severity of asthma and diagnosis using the same defined method can help reduce this variation. Also, it is important to ensure that the patients analyzed have no other underlying airway disease or other comorbidity. Accessing samples from pure patient populations can be challenging; however it can greatly decrease the number of false positives within the resulting microarray dataset.

## 12. Accessing Previous Microarray Study Data

In 2002, the Nature family of journals adopted the minimum information about microarray experiments (MIAMEs) standard (developed by the Microarray Gene Expression Data Society (MGED) [70]), making it mandatory that all microarray data (including annotations) used in publishable research must be deposited into an acceptable public repository (NCBI Gene Expression Omnibus (GEO) [71], ArrayExpress [72], or Center Information Biology Gene Expression Database (CIBEX) [73], prior to the submission of a manuscript [74]. There are six key elements within the MIAME guidelines which authors must provide:

1. raw data for each microarray (e.g., cel files),
2. the processed normalized data,
3. annotation of the samples used to conduct the microarrays (treatment, cell types, etc.),
4. the experimental design,
5. annotation of the array itself (coordinates of probes and their sequences),
6. methods of normalizing the data to obtain the processed data.

Over the years, many other journals have also adopted the MIAME standard, turning GEO (http://www.ncbi.nlm.nih.gov/geo/) into a free microarray database containing ~20,000 microarray studies to date [75]. Because this information is freely available, researchers now have the opportunity to design specific questions and search for a previous microarray project to help narrow down the list of candidate genes involved with their function of interest; the GEO accession numbers for all microarrays' studies discussed in this review are highlighted in Table 4. However, not all the scientific community believe that the MIAME guidelines are beneficial. A number of critics have expressed the view that forcing groups to disclose their microarray data upon publishing has led to many to groups simply not publishing their findings. Whether this restriction is resulting in a biased reporting of the application of microarrays in research is yet to be determined. Another issue is the inability of researchers to repeat the analysis of published expression microarrays in MIAME abiding journals. This problem was recently discussed in a paper by Ioannidis et al. [76]. In this study Ioannidis and colleagues attempted to replicate the data analyses in 18 articles on microarray-based gene expression published in Nature Genetics in 2005-2006. Of the 18 articles ten could not be reproduced [76]. The main reasons for this were data unavailability (even though these articles were published under the MIAME guidelines) and incomplete data annotation not abiding by the MIAME guidelines [76]. Based on this study and a number of reviews it is clearly evident that MIAME provides a method of allowing researchers to share and scrutinise microarray based data by providing the necessary information; however it is only as effective as the enforcement that journals which abide by these guidelines impose on researchers to make sure that they follow them correctly [76, 77].

## 13. Conclusion

Microarrays have significantly increased our understanding of the genes and cell types involved with asthma. Although the use of microarrays in asthma research is still at an early stage, it has helped confirm the results of previous studies and has identified a number of novel genes which warrant further, investigation. As the price of microarrays decreases and the technology advances further the use of microarrays in asthma-related research will expand and may provide exciting new insights into the genetic regulation of this complex pathological process.

## Figures and Tables

**Table 1:** Databases used in Affymetrix microarray annotation.

Database	Description		Website		References
Expressed Sequence Tag Database (dbEST)	Division of GenBank that contains “single-pass" cDNA sequences (only sequenced once), or “Expressed Sequence Tags"	http://www.ncbi.nlm.nih.gov/dbEST/			[78]
The Institute for Genomic Research (TIGR)	Constructed by clustering, then assembling expressed sequence tag (EST) and annotated gene sequences from GenBank	http://compbio.dfci.harvard.edu/tgi/			[79]
UniGene Build	Contains transcript sequence information including: protein similarities, gene expression, cDNA clone reagents, and genomic location	http://www.ncbi.nlm.nih.gov/unigene/			[80]
GenBank	Annotated collection of all publicly available DNA sequences	http://www.ncbi.nlm.nih.gov/genbank/			[81]
The Reference Sequence (RefSeq)	Contains nonredundant, and well-annotated genomic DNA, transcripts, and protein sequences	http://www.ncbi.nlm.nih.gov/RefSeq/			[82]

**Table 2:** Asthma-related phenotypes that result from aberrant expression of splice variants.

Symbol	Gene Name	Phenotypes	Description	Reference
NPSR1	neuropeptide S receptor 1	IgE levels and Asthma	Isoform B over expressed in asthmatic ASM cells	[15]
IL-4	interleukin-4	Asthma	Alternatively spliced variants of IL-4 mRNA are differently expressed in patients with bronchial asthma	[58, 59]
COX-1	cytochrome c oxidase assembly homolog (yeast)	Asthma	Alternatively spliced variants of COX-1 mRNA are differently expressed in patients with bronchial asthma	[60]

**Table 3:** List of a number companies currently providing miRNA microarray technology.

Company	Microarray	Link
Ambion	*mir*Vana miRNA bioarrays V2	http://www.ambion.com/
Agilent Technologies	Human miRNA Microarray Release 16.0	http://www.genomics.agilent.com/
Affymetrix	GeneChip miRNA 2.0 Array	http://www.affymetrix.com/
Exiqon	miRCURY LNA*™* microRNA Array	http://www.exiqon.com/
Invitrogen	NCode*™* Human miRNA Microarray V3	http://products.invitrogen.com/
LC Sciences	V17.0 Human microRNA Microarray	http://www.lcsciences.com/
Miltenyi Biotec	miRXplore*™* Microarray Kits	http://www.miltenyibiotec.com/
System biosciences	miRNome MicroRNA Profilers	http://www.systembio.com/

**Table 4:** The GEO accession number for microarray studies conducted on asthma.

Year	Title	Array	GEO accession number	Reference
Smooth muscle cells				

2001	Interleukin-13 induces dramatically different transcriptional programs in three human airway cell types	Affymetrix Hugene FL	n/a	[21]
2004	Effects of interleukin-1 [beta], interleukin-13, and transforming growth factor-[beta] on gene expression in human airway smooth muscle using gene microarrays	Affymetrix GeneChip 95A	n/a	[23]
2005	The effect of IL13 and IL13R130Q, a naturally occurring IL13 polymorphism, on the gene expression of human airway smooth muscle cells	8159 human gene cDNA clones from Research Genetics (IMAGE consortium, Huntsville, AL), Incyte Genomics	n/a	[24]
2007	1*α*,25-Dihydroxy-vitamin D_3_ stimulation of bronchial smooth muscle cells induces autocrine, contractility, and remodeling processes	Human Genome U133 Plus 2.0 GeneChip arrays	GSE5145	[26]
2009	Glucocorticoid- and protein kinase A-dependent transcriptome regulation in airway smooth muscle	Affymetrix Human U133A DNA microarrays	GSE13168	[25]
2010	MicroRNA expression in human airway smooth muscle cells: role of miR-25 in regulation of airway smooth muscle phenotype	*mir*Vana miRNA bioarrays V2 (Ambion)	GSE16587 GSE16586	[67]

Epithelial cells				

2002	Analysis of novel disease-related genes in bronchial asthma	Affymetrix Hugene FL	n/a	[34]
2003	Respiratory syncytial virus infection activates STAT signaling in human epithelial cells	Affymetrix Hugene FL	n/a	[35]
2006	Induction of the plasminogen activator system by mechanical stimulation of human bronchial epithelial cells	Affymetrix Human 133A DNA microarrays	n/a	[36]
2007	IL-13 and epidermal growth factor receptor have critical but distinct roles in epithelial cell mucin production	UCSF 9Hs Human 23K v.2 Oligo Array	GSE4804	[37]
2007	Genomewide profiling identifies epithelial cell genes associated with asthma and with treatment response to corticosteroids	Human Genome U133 Plus 2.0 GeneChip arrays	GSE4302	[41]
2009	Airway epithelial cells regulate the functional phenotype of locally differentiating dendritic cells: implications for the pathogenesis of infectious and allergic airway disease	Human Genome U133 Plus 2.0 GeneChip arrays	GSE12773	[38]
2009	T-helper type 2-driven inflammation defines major subphenotypes of asthma	Human Genome U133 Plus 2.0 GeneChip arrays	GSE4302	[39]
2010	Rhinovirus-induced modulation of gene expression in bronchial epithelial cells from subjects with asthma	Human Genome Focus GeneChip Array Human Genome U133 Plus 2.0 GeneChip arrays	GSE13396	[40]
2010	Transglutaminase 2, a novel regulator of eicosanoid production in asthma revealed by genomewide expression profiling of distinct asthma phenotypes	Affymetrix Human U133A DNA microarrays Human Genome U133 Plus 2.0 GeneChip arrays	GSE13785	[83]
2010	Decreased fibronectin production significantly contributes to dysregulated repair of asthmatic epithelium	Affymetrix Human 133A DNA microarrays	GSE18965	[42]

Mast Cells				

2001	Gene expression screening of human mast cells and eosinophils using high-density oligonucleotide probe arrays: abundant expression of major basic protein in mast cells	Affymetrix GeneChip 95A	n/a	[51]
2005	Amphiregulin expression in human mast cells and its effect on the primary human lung fibroblasts	Affymetrix Genechip Human Genome U133	n/a	[52]
2009	Human mast cells synthesize and release angiogenin, a member of the ribonuclease A (RNase A) superfamily	NIAID (human sequence chip series “sa”) and consist of 13,971 oligonucleotides, synthesized by Qiagen Operon Inc. (Valencia, CA, USA)	n/a	[53]

Tissue				

2004	Functional classes of bronchial mucosa genes that are differentially expressed in asthma	Affymetrix GeneChip 95A	GSE15823	[22]
2010	Gene transcription changes in asthmatic chronic rhinosinusitis with nasal polyps and comparison to those in atopic dermatitis	Affymetrix HuExon 1.0 ST	GSE5667	[63]

GEO: NCBI Gene Expression Omnibus.

n/a: microarray data not submitted to a database or not stated in paper.
